# Improvement of optic nerve pial blood supply visualized through indocyanine green videoangiography after resection of a tuberculum sellae meningioma: 2-dimensional operative video

**DOI:** 10.3171/2021.10.FOCVID21155

**Published:** 2022-01-01

**Authors:** Guilherme H. W. Ceccato, Júlia S. de Oliveira, Pedro H. dos Santos Neto, Nick D. Carvalho, Vinicius N. Coelho, Hugo A. Hasegawa, Sergio L. Sprengel, Marcio S. Rassi, Luis A. B. Borba

**Affiliations:** 1 Department of Neurosurgery, Mackenzie Evangelical University Hospital (HUEM), Curitiba, PR;; 2 Department of Neurosurgery, Federal University of Paraná, Curitiba, PR; and; 3 Department of Neurosurgery, A.C. Camargo Cancer Center, São Paulo, SP, Brazil

**Keywords:** optic nerve, indocyanine green, meningioma, visual field, ischemia

## Abstract

Ischemia of the optic nerve (ON) is an important cause of visual field deficit provoked by tuberculum sellae (TS) meningiomas. Indocyanine green (ICG) videoangiography could provide prognostic information. Moreover, it allows new insight into the pathophysiology of visual disturbance. The authors present the case of a 48-year-old woman with visual field impairment. Magnetic resonance imaging (MRI) depicted a lesion highly suggestive of a TS meningioma. Following microsurgical resection, ICG videoangiography demonstrated improvement of right ON pial blood supply. In this case, there was one lesion causing visual impairment through both direct compression over the left ON and ischemia to the right nerve.

The video can be found here: https://stream.cadmore.media/r10.3171/2021.10.FOCVID21155

## Transcript

This video demonstrates the improvement of optic nerve pial blood supply visualized through indocyanine green videoangiography after resection of a tuberculum sellae meningioma.

### 0:31 Clinical History.

The patient was 48-year-old female presenting with a history of progressive headache, galactorrhea, and mild visual field impairment, as well recurrent high serum prolactin levels despite treatment with cabergoline.

### 0:45 Preoperative Visual Field Examination.

Preoperative visual field examination demonstrated both sides compromised, but with slightly greater and more diffuse deficit on the right eye.

### 0:53 Preoperative Imaging.

Preoperative MRI demonstrated a mass highly suggestive of a tuberculum sellae meningioma. And there was no evidence of optic canal invasion. Here we can better see the lesion that measured around 17 × 10 mm. Now we can observe the tumor in the three orthogonal planes. And here the displacement of the left optic nerve, with the right nerve just touched by the tumor, which is interesting considering that the visual field examination of the right nerve was slightly worse regarding the left. And considering worsening of the symptoms, microsurgical resection was indicated.

### 1:25 Positioning, Skin Incision, and Soft-Tissue Dissection.

The patient was placed supine with the head rotated 30° to contralateral side and slightly deflected. Skin incision running from in front of tragus to the contralateral midpupillary line behind the hairline is employed. During elevation of the cutaneous flap, it is important to take care to preserve at least the frontal branch of superficial temporal artery. It is important to prepare a wide pedicled pericranial flap to help later in closure of skull base defects. And now we can observe the flap prepared in this case. Temporalis muscle is dissected using a subfascial technique to preserve the frontalis branch of facial nerve. And then the soft tissues close to orbital rim are further elevated in a subperiosteal fashion. Following dissection of temporalis muscle from the underlying bone, it is reflected inferiorly, and a fronto-orbital craniotomy is performed.

### 2:20 Craniotomy 3D Models.

In this 3D model we can observe the bone flap. An important burr hole is at MacCarty keyhole, exposing both orbit and anterior fossa, and the last osteotomy to release the bone flap is through orbital roof accessed using this point. As we can observe from this inner perspective, additional removal of orbital roof to increase space is performed after taking out the main bone flap, and this piece can be put back in the end during reconstruction. Now we can observe the exposure through the craniotomy window and demonstrate opening of frontal sinus, which must be cranialized and previous harvested pericranial flap can help during its closure. And now we can observe exposure of region of tuberculum sellae.

### 3:05 Tumor Exposure.

Following Sylvian fissure dissection, the tumor, right optic nerve, internal carotid artery, and olfactory tract were exposed. And progressively the optic chiasm is being demonstrated. Since the beginning it is important to dissect preserving arachnoid planes. Now it is possible to demonstrate a branch of a superior hypophyseal artery running toward right optic nerve compressed by the tumor.

### 3:34 Indocyanine Green Videoangiography Before Resection.

Before starting to resect the lesion, an ICG videoangiography was performed.^1^ And here we have the view of surgical field. A bolus injection of 25 mg of indocyanine green was applied in a peripheral vein diluted in 5 ml of distillate water and the fluorescence mode of microscope was activated. We observed initially the filling of the internal carotid artery and then the intracranial structures, as the meningioma, and observed just a modest filling of the optic nerve pial vessels. After careful inspection it is possible to identify a pattern of filling of the superior pial network in a horizontal fashion apparently by collateral flow from ophthalmic artery anteriorly and chiasmatic vessels posteriorly. As the blood does not seem to come from inside the optic nerve itself, but apparently come gradually filling the anterior and posterior extremities of the pial vessels.

### 4:23 Steps for Tumor Resection.

Following the ICG videoangiography the lesion started to be removed. Optic nerve is initially released from its arachnoid adhesions to allow safer manipulation of the mass during its resection. Tumor removal starts through its base, coagulating its attachment to the region around tuberculum sellae, to from the beginning attack its blood supply. Progressively the whole extent of the attachment of the mass is being coagulated and sectioned under constant irrigation. Here advancing more to the left side. And now the attachment is approached deeper. The removal of the tumor should respect the arachnoid planes around it to preserve the surrounding neurovascular structures, as we can observe the arachnoid membranes in the inferior and deeper corner of dissection of the mass.^2^ And here, more from the left side of tumor attachment is coagulated. Now the inferior corner of the right side of tumor attachment is finally sectioned releasing this side, exposing the arachnoid membranes deeper. And here a superior hypophyseal artery running inside this arachnoid is demonstrated, vessels that must be preserved when dissecting around optic apparatus, as they are an important source of blood supply to optic nerves.^3–6^ Then dissection progress more to the contralateral side, coagulating and sectioning the left side of tumor attachment, and gradually we see exposed the left optic nerve and contralateral internal carotid artery, with attention to preserve the arachnoid planes during dissection. Now we are dissecting the left side of tumor displacing left optic nerve, releasing the arachnoid adhesions of this side of the mass. After circumferential dissection the mass can be removed en bloc, demonstrating the side of tumor attachment while removing it. And here we can observe preservation of the previous identified superior hypophyseal artery inside arachnoid membranes.^2^ Now we are dissecting around the left optic nerve and expose the course of internal carotid artery. Drilling of the region of tumor attachment is performed. Here a residual tumor is identified in the anterior wall of sella. And then it is successfully dissected and removed. Intraoperatively, invasion of the optic canal was not identified from neither side, and it was not shown in the preoperative images either; therefore, the optic canal was not opened in this case. We look for eventual other tumor remnants and remove small pieces. And proceed to additional drilling and coagulation of the area of tumor attachment to achieve a Simpson grade I resection.

### 7:08 Indocyanine Green Videoangiography After Resection.

Following tumor removal, we performed a new ICG videoangiography, with the same methodology used before. We observe initially filling of internal carotid artery and then the intracranial structures, as the previous identified superior hypophyseal artery running between optic nerves. We can observe a faster filling of pial vessels of right optic nerve and observe an apparent engorgement of the superior pial network, which seemed to fill supplied by vessels from inside optic nerve, in a vertical fashion. We hypothesized that the tumor compromised vessels supplying the inferior pial network, and after tumor resection the blood supply of this network improved and consequently the intrinsic optic nerve blood supply, which in its turn increased blood flow to the superior pial network.^7^

### 7:57 Comparison Between Pre- and Postoperative ICG Videoangiography.

Now we have both ICG videoangiographhy before and after resection and can observe the improvement of optic nerve pial blood supply following tumor removal. We put the previous both videos side by side a little bit accelerated for the purpose of comparison; however, we can better see the difference here, through these color maps according to the fluorescence signal intensity. We captured a sequence of still images from the native ICG video with intervals of 1 second using the software GOM Player and then converted the grayscale images to a color map based in a look-up table of 16 colors using the software ImageJ.

Here we can better see the difference of blood flow in the superior pial network. We considered as the time point zero when the related signal intensity of the internal carotid artery measured in the ImageJ reached 100, to standardize the comparison.

### 8:55 Quantitative Comparison Between ICG Videoangiography Before and After Resection.

Using the software ImageJ, we selected a region of interest over right optic nerve and built a chart of fluorescence signal intensity. To facilitate the comparative, the baseline values obtained by the region of interest were discounted to normalize comparison and allow both curves to start from signal intensity zero. We can observe significant improvement of signal intensity, what means blood supply, following resection.

### 9:19 Postoperative Imaging.

Postoperative MRI demonstrated complete tumor resection, relieving the compressive effect of the lesion, as we can observe in the FIESTA sequence, as well in these postcontrast T1 weighted images. We see the axial and now coronal and sagittal cuts. Now we have a comparative between before and after resection exposing resolution of compressive effect over optic apparatus, especially over the left optic nerve.

### 9:50 Postoperative Visual Field Examination.

Considering the visual field examination, we observed both eyes’ improvement. And here we have the Humphrey perimetry. And now the comparison between the visual field examination through the same method, demonstrating both eyes’ improvement, with a more significant improvement of the right eye.

### 10:08 Outcome.

Pathology confirmed the lesion to be a meningioma, and patient presented improvement of symptoms and no new neurological deficit on follow-up.

## Disclosures

The authors report no conflict of interest concerning the materials or methods used in this study or the findings specified in this publication.

## Author Contributions

Primary surgeon: Borba. Assistant surgeon: de Oliveira, Hasegawa, Sprengel. Editing and drafting the video and abstract: Borba, Ceccato, dos Santos Neto, Coelho, Rassi. Critically revising the work: Borba, Ceccato, dos Santos Neto, Coelho, Sprengel, Rassi. Reviewed submitted version of the work: Borba, Ceccato, de Oliveira, de Carvalho, Coelho, Rassi. Approved the final version of the work on behalf of all authors: Borba. Supervision: Ceccato, de Oliveira, Coelho, Sprengel. Conception: Ceccato.
